# A homozygous *STIM1* mutation impairs store-operated calcium entry and natural killer cell effector function without clinical immunodeficiency

**DOI:** 10.1016/j.jaci.2015.08.051

**Published:** 2016-03

**Authors:** David A. Parry, Tim D. Holmes, Nikita Gamper, Walid El-Sayed, Nishani T. Hettiarachchi, Mushtaq Ahmed, Graham P. Cook, Clare V. Logan, Colin A. Johnson, Shelagh Joss, Chris Peers, Katrina Prescott, Sinisa Savic, Chris F. Inglehearn, Alan J. Mighell

**Affiliations:** a Section of Ophthalmology and Neuroscience, School of Medicine, St James's University Hospital, University of Leeds, Leeds, United Kingdom; b Section of Genetics, School of Medicine, St James's University Hospital, University of Leeds, Leeds, United Kingdom; c Leeds Institute of Cancer and Pathology, School of Medicine, St James's University Hospital, University of Leeds, Leeds, United Kingdom; d Center for Infectious Medicine, Karolinska University Hospital, Stockholm, Sweden; e School of Biomedical Sciences, University of Leeds, Leeds, United Kingdom; f School of Dentistry, University of Leeds, Leeds, United Kingdom; g Oral Biology Department, Dental Collage, Gulf Medical University, Ajman, United Arab Emirates; h Division of Cardiovascular and Diabetes Research, School of Medicine, University of Leeds, Leeds, United Kingdom; i Clinical Genetics, Leeds Teaching Hospitals NHS Trust, Chapel Allerton Hospital, Leeds, United Kingdom; j Clinical Genetics, Southern General Hospital, Glasgow, United Kingdom; k Department of Clinical Immunology and Allergy, Leeds Teaching Hospitals NHS Trust, St James's University Hospital, University of Leeds, Leeds, United Kingdom

To the Editor:

Stromal interaction molecule 1 (STIM1) is a transmembrane protein pivotal to store-operated calcium entry (SOCE) that localizes to either the cell or endoplasmic reticulum (ER) membranes, with the N-terminus in either the extracellular space or the ER, respectively. Plasma membrane ORAI calcium release–activated calcium modulator 1 (ORAI1) Ca^2+^ channels are activated by STIM1. Families previously described with recessive *STIM1* mutations (MIM #612783) had life-threatening viral, bacterial, and fungal infections; developmental myopathy; hypohidrosis; and amelogenesis imperfecta (AI; generalized developmental enamel abnormalities).^1^, ^2^, ^3^ We investigated a consanguineous family, segregating a novel syndrome of recessive AI and hypohidrosis by using autozygosity mapping and clonal sequencing. A homozygous rare missense mutation in *STIM1* (p.L74P) in the EF-hand domain was identified (see the Methods and Results sections in this article's Online Repository at www.jacionline.org).

The family was re-evaluated, with particular attention paid to features associated with recessive *STIM1* mutations (Table I and see Table E1, Table E2, Table E3 in this article's Online Repository at www.jacionline.org). The 2 affected cousins (18 and 11 years old, respectively) did not have overt clinical immunodeficiency. Further evaluation of their immune systems showed a normal immunoglobulin profile with an adequate specific antibody response to both nonlive (pneumococcus, tetanus and, Hib) and live (mumps, measles, and rubella) vaccinations. In addition, both subjects had detectable IgG against varicella zoster virus after a previous uncomplicated primary infection. The younger cousin was also found to have IgG against EBV viral capsid antigen, suggesting previous exposure, but neither showed any evidence of acute infection or previous exposure to cytomegalovirus.

Lymphocyte studies showed stable CD8 T-cell depletion in the older affected subject only. Other lymphocyte subsets, including CD4 T, natural killer (NK), and B cells, were within the normal range (Table I). However, despite normal PHA and anti-CD3 simulation responses, T-lymphocyte and NK cell SOCE was grossly abnormal, which is consistent with disruption of the Ca^2+^-binding EF-hand and in keeping with previous reports for recessive *STIM1* mutations (see Fig E1, *A*, in this article's Online Repository at www.jacionline.org).^1^, ^2^, ^3^ The defect in NK cell SOCE was associated with impaired NK cell effector function, as shown by assays of granule exocytosis and intracellular IFN-γ production in response to K562 tumor cells (see Fig E1, *B*). After recently published mouse studies, which confirmed the importance of STIM1 to neutrophil SOCE and associated functions,^4^ we also evaluated neutrophil function. This was found to be within normal limits.

Despite abnormal immune system SOCE, the affected subjects in this case appear to be able to compensate for this deficit and avoid overt immunodeficiency. It is possible that the relative preservation of T-cell function might compensate for NK cell dysfunction. Neither might yet have encountered a pathogen that would expose this particular immune system limitation (see Table E2). An ability to mount a partial response to viral infections was reported in a family with clinical immunodeficiency and a history of viral infections caused by a homozygous missense R429C change affecting the STIM1 cytoplasmic domain.^2^ A mouse model characterized by conditional knockout of *Stim1* and *Stim2* in both CD4^+^ and CD8^+^ T cells has recently provided further insight into the importance of Stim1 in immune system development and virus-specific memory and recall responses, which prevent acute viral infections from becoming chronic.^5^

Recessive *STIM1* mutations can be associated with other immune dysregulations, including autoimmune disease. The older cousin had a transient episode of idiopathic thrombocytopenic purpura when 2 years old that might have been unrelated to the *STIM1* mutations. There were no other clinical or serologic markers consistent with autoimmune disease, and regulatory T-cell numbers were normal.

Both cousins were intolerant of warm environments and aware of their inability to sweat normally. This limited the older cousin's ability to participate in sport. There was no clinical or serologic evidence of myopathy. This is in contrast to other recessive *STIM1* mutations and also to dominant *STIM1* mutations affecting the EF-hand that cause tubular aggregate myopathy (MIM #160565).^6^ Hypomineralized AI affected the primary and secondary dentitions of both affected cousins (see Fig E2 in this article's Online Repository at www.jacionline.org), which is in keeping with reports of other recessive *STIM1* mutations. The cousins were physically small (height, weight, and head circumference <0.4th percentile) when assessed at 18 years and 9 years, 10 months of age, respectively. Without comparable data from other subjects with recessive *STIM1* mutations, it is unclear whether this is a cosegregating feature.

The L74P STIM1 change within the EF-hand domain precedes the first Ca^2+^-binding aspartate residue by 2 amino acids (see Fig E2) and therefore might be expected to distort the Ca^2+^-binding region of the protein. Therefore we compared the response of mutant YFP-STIM1 (L74P) with the depletion of Ca^2+^ stores after thapsigargin or cyclopiazonic acid (CPA) treatment with that of wild-type YFP-STIM1 and the previously published EF-hand mutant^7^ YFP-STIM1 (D76A, see Fig E3 in this article's Online Repository at www.jacionline.org).

Using total internal reflection fluorescence microscopy (TIRFM), we replicated previous observations that wild-type YFP-STIM1 relocalizes to puncta proximal to the plasma membrane after treatment of transfected HEK293 cells with 2 μmol/L thapsigargin to deplete ER Ca^2+^ stores through sarcoendoplasmic reticulum calcium transport ATPase (SERCA) inhibition (see Fig E3, *A*). The EF-hand mutant YFP-STIM1 (D76 A) was present in these puncta before thapsigargin treatment, with no observable response to thapsigargin (see Fig E3, *A*). Similarly, mutant YFP-STIM1 (L74P) showed no response to thapsigargin but also appeared to form constitutive puncta, which was less distinct in appearance than that for the D76A mutant (see Fig E3).

We compared Ca^2+^ fluctuations in HEK293 cells transfected with ORAI-CFP and either wild-type YFP-STIM1, mutant YFP-STIM1 (D76A), or mutant YFP-STIM1 (L74P; see Fig E3, *B* and *C*). Both YFP-STIM1 (D76A) and YFP-STIM1 (L74P) transfected cells had increased basal Ca^2+^ concentrations compared with wild-type YFP-STIM1 and reduced peak and integral responses to CPA-induced SERCA inhibition (see Fig E3, *B* and *C*). However, in contrast to the EF-hand mutant YFP-STIM1 (D76A), YFP-STIM1 (L74P) did not demonstrate reduced SOCE after CPA washout and Ca^2+^ restoration, suggesting that the previously reported desensitization of SOCE observed with the YFP-STIM1 (D76A) mutant does not occur with the YFP-STIM1 (L74P) mutant form. Therefore the L74P mutation appears to result in a distinct molecular phenotype compared with the loss of function observed in immunodeficient patients and the constitutive activation observed in patients with myopathy.

This study is the first to report recessive *STIM1* mutations in patients presenting with AI and hypohidrosis without overt clinical immunodeficiency or myopathy. Clinical immunologic investigations were consistent with abnormal NK cell and T-lymphocyte function that might be expected to be associated with ongoing clinical immunodeficiency. However, despite severely abnormal SOCE, this was not the case in these patients. Missense mutations affecting the EF-hand can have very different clinical phenotypes with respect to the immune system, muscle, sweating, and enamel formation. This has important implications for clinical evaluation, as well as understanding the biological functions of STIM1.

## Figures and Tables

**Fig E1:**
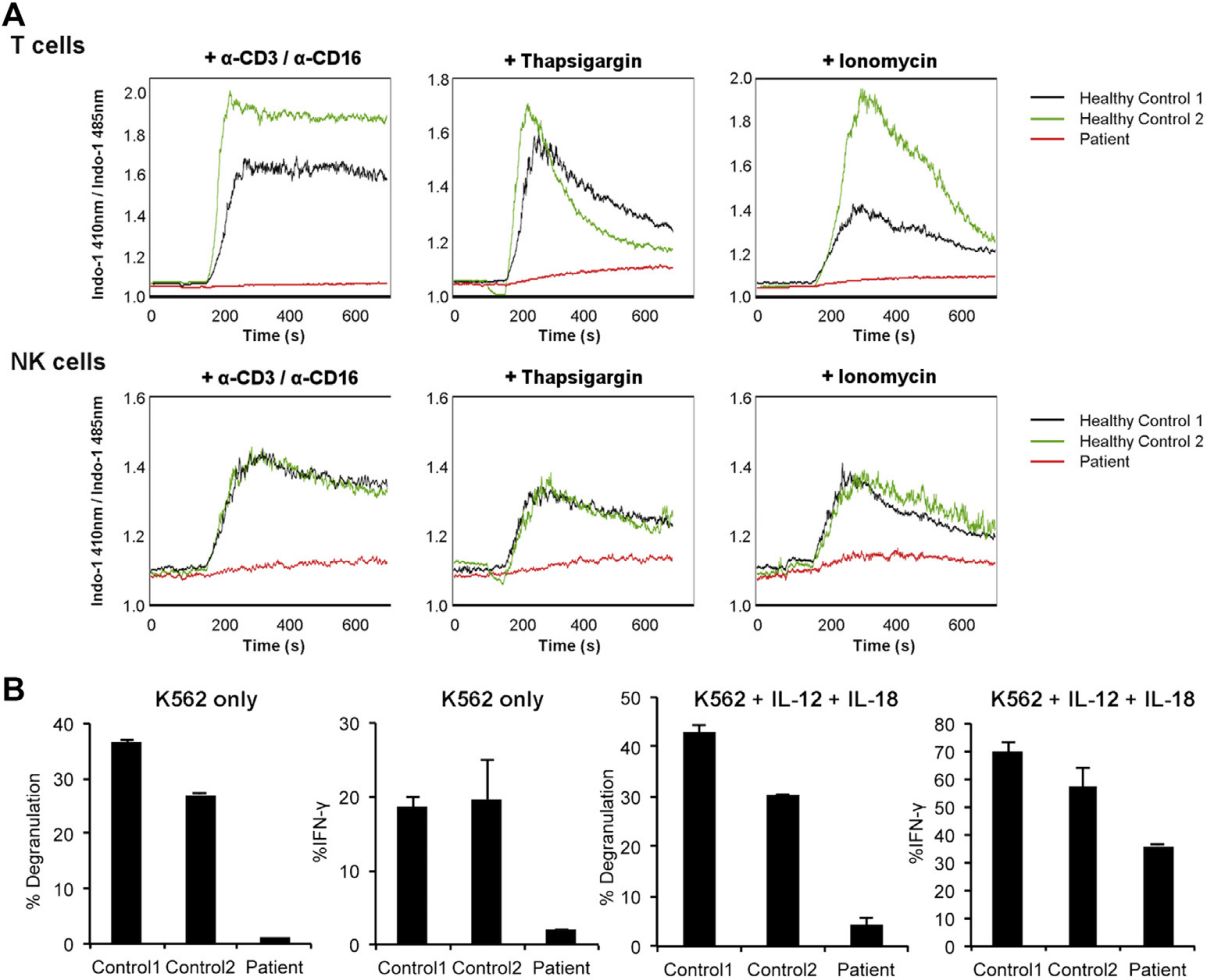
Defective SOCE and impaired NK cell function in STIM1-Leu74Pro patients' cells. **A,** Calcium flux in lymphocytes after anti-CD3/anti-CD16, 1 μmol/L thapsigargin, or 500 nmol/L ionomycin administration. **B,** Granule exocytosis and IFN-γ production of purified NK cells after stimulation with K562 tumor target cells alone or with IL-12 and IL-18. Results are representative of 2 experiments performed in duplicate and corrected for unstimulated control values.

**Fig E2:**
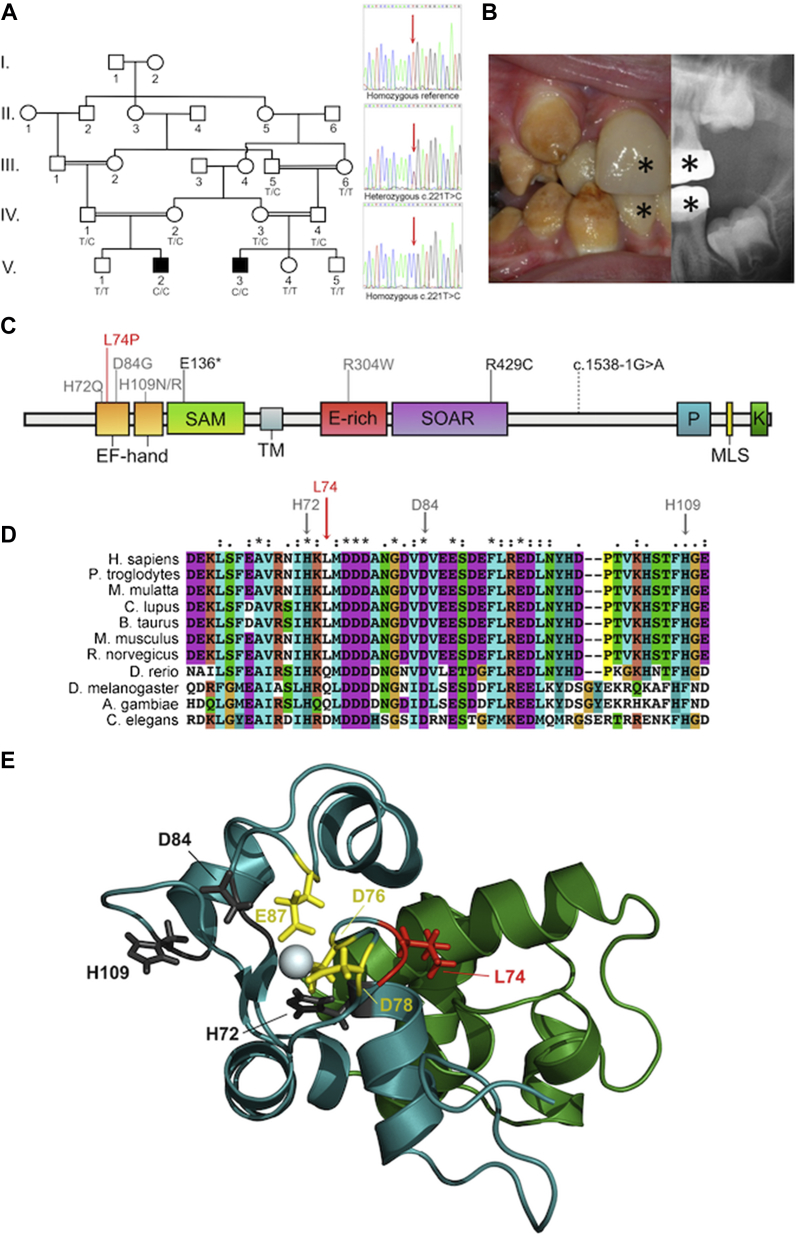
Hypomineralized AI as the presenting feature in a family with STIM1 L74P change. **A,** Pedigree of the consanguineous family investigated. The 2 affected cousins with AI and hypohidrosis are shaded *black*. Genotypes of the c.221T>C variant are indicated underneath each family member available for sequencing. Representative electropherograms are shown alongside the pedigree. **B,** The hypomineralized AI was characterized by opaque discolored enamel on clinical examination, with radiographs of unerupted teeth consistent with a near-normal volume of enamel and a clear difference in radiodensity between enamel and dentine. *Teeth that have been restored. **C,** Schematic illustration of STIM1 protein showing the domain structure. Positions of the AI and hypohidrosis-associated L74P mutation *(red)*, dominant TAM or Stormorken syndrome mutations *(grey)*, and recessive syndromic immunodeficiency mutations *(black)* are indicated above the protein. *E-rich*, Glutamate-rich region; *K*, lysine-rich region; *MLS*, microtubule tip localization signal; *P*, proline/serine-rich region; *SAM*, sterile α-motif domain; *SOAR*, STIM1 Orai1-activating region; *TM*, transmembrane domain. **D,** Alignment of STIM1 EF-hand orthologous protein sequences. Although p.L74 is conserved in mammals, it is not as strongly conserved as amino acids mutated in dominant TAM. **E,** NMR structure of STIM1.^E7^ L74 is shown in *red*, TAM mutations are shown in dark gray, and Ca^2+^ binding residues, mutation of which cause constitutive STIM1 activation, are shown in yellow. Substitution of leucine 74 for proline is anticipated to distort the EF-hand loop, interfering with conformational changes in the presence/absence of Ca^2+^.

**Fig E3:**
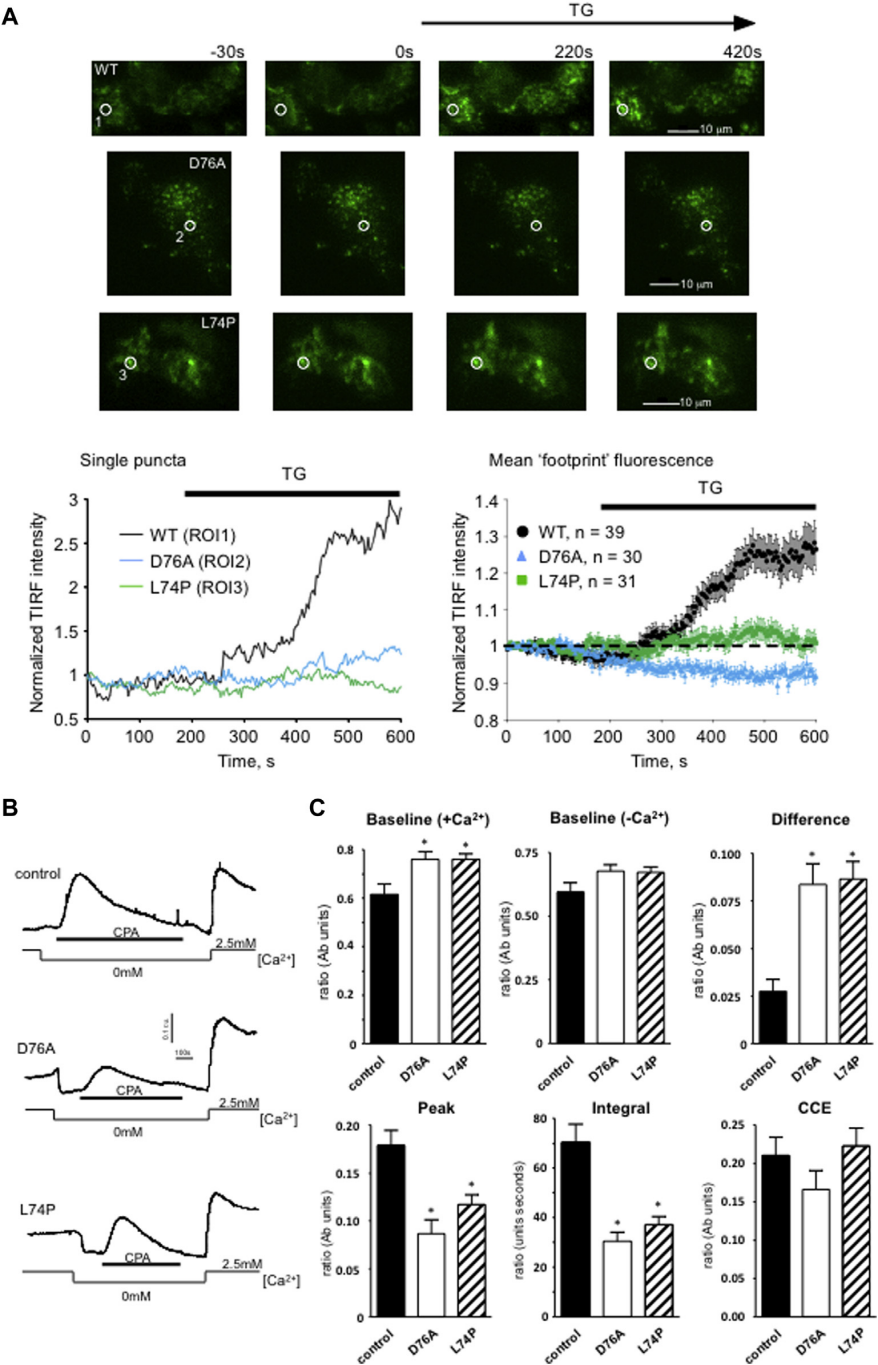
STIM1 localization and Ca^2+^ flux in cells transfected with STIM1 constructs. **A,** TIRFM of HEK293 cells transfected with either wild-type *(WT)*, D76 A mutant, or L74P mutant YFP-STIM1 after treatment with 2 μmol/L thapsigargin to deplete ER Ca^2+^ stores. The *graph on the bottom left* shows changes in TIRF fluorescence within single puncta areas indicated by *white circles* on the images (ROI1-3). The *graph on the bottom right* shows average footprint fluorescence for cells transfected with WT STIM1 (n = 39), D76 A (n = 30), or L74P (n = 31) constructs. **B,** Representative recordings of cytosolic calcium ([Ca^2+^]_i_) made in HEK293 cells doubly transfected with ORAI1-CFP and either WT (n = 12), D76 A (n = 12), or L74P (n = 13) STIM1-YFP constructs. **C,** Bar graphs indicating mean ± SEM [Ca^2+^]_i_ relating to the presence of extracellular Ca^2+^ or SERCA inhibition by CPA. *Top row*, Mean baseline Ca^2+^ levels in the presence and absence of extracellular Ca^2+^. *Bottom row*, Peak responses to CPA, integral of the CPA-evoked response, and peak value of capacitative Ca^2+^ entry *(CCE)*. *P < .01 compared with control with control values (ANOVA).

**Table E1:** Additional clinical features of the 2 subjects with homozygous *STIM1* c.221T>C mutations

Feature	V3	V2
Birth and neonatal period	Full-term (2.3 kg) by using forceps for fetal distress	Emergency cesarean section because of fetal decelerations at 36/40 wk Birth weight, 1.94 kg (<3rd percentile) Apgar score, 9 at 1 and 5 minutes, respectively Special care baby unit for 1 mo, establishing feeds with nasogastric tube feeds for the first 2 wk During this time, there was 1 episode of unexplained fever. Unexplained neonatal hypercalcemia settled spontaneously.
Nails and hair	Normal	Normal
Dysmorphic features	None	None
Other medical history	Asthma diagnosed in infancy Evaluated in infancy for cystic fibrosis (negative) after repeated chest infections At age 17 y, had a spontaneous pneumothorax of the left lung requiring pleurodesis Four apical bullae were found on imaging. Five months later, he had a right pneumothorax secondary to an apical bulla also requiring pleurodesis. At aged 18 y, V3 was evaluated with regard to macular pigmentation and bilateral drusen on both maculae, and mild congenital lens opacities were identified.	Asthma diagnosed in infancy Eczema Generalized problems with increased leg fatigability and clumsiness Tight Achilles tendons of unknown cause Bilateral pes cavus Hypermobility in upper limbs
Allergies	Allergic to red food coloring	None

**Table E2:** Summary of clinical immunologic data in subjects with either homozygous or heterozygous STIM1 c.221T>C mutations

Feature	VI:2		V:1		V:2	VI:3	
Homozygous c.221T>C		Heterozygous c.221T>C		Heterozygous c.221T>C	Homozygous c.221T>C	
Year of evaluation	2011	2014	2011	2014	2011	2012	2013
Bacterial antibodies							
Pneumococcal (μg/mL)	209.0	−	101.0	−	82.2	181.0	−
Tetanus (IU/mL)	0.890	−	5.320	−	0.960	0.620	−
*Haemophilus* species (μg/mL)	0.230	−	0.240	−	<0.110	0.430	−
Viral antibodies							
HSV IgG	ND	ND	−	+ve	−	−	−
VZV IgG	+ve	+ve	−	+ve	−	+ve	−
CMV IgM	ND	−	−	ND	−	−	−
CMV IgG	ND	ND	−	+ve	−	ND	−
EBV VCA IgM	ND	−	−	ND	−	−	−
EBV VCA IgG	ND	−	−	+ve	−	+ve	−
Measles IgG	−	+ve	−	+ve	−	−	−
Mumps IgG	−	+ve	−	+ve	−	+ve	−
Rubella IgG	−	+ve	−	+ve	−	+ve	−
Viral PCR							
EBV	−	ND	−	−	−	−	−
CMV	−	ND	−	−	−	−	−
Adenovirus	−	ND	−	−	−	−	−
Lymphocytes							
Total (×10^9^/L [1.00-2.80])	1.50	1.32	2.30	2.35	2.64	2.34	2.20
CD4/CD8 (1.07-1.87)	15.29*	**10.58**	**4.62**	**5.87**	**3.62**	**1.86**	**1.9**
CD3 (absolute; × 10^9^/L [0.700-2.100])	0.921	0.949	1.365	1.605	1.968	1.504	1.457
CD8 (absolute; × 10^9^/L [0.200-0.900])	**0.055**	**0.080**	0.236	0.231	0.415	0.488	0.489
NK (absolute; × 10^9^/L [0.090-0.600])	0.238	0.191	0.581	0.449	0.252	0.252	0.152
CD4 (absolute; × 10^9^/L [0.300-1.400])	0.841	0.846	1.091	1.355	1.502	0.908	0.929
CD19 (absolute; × 10^9^/L [0.100-0.500])	0.258	0.127	0.245	0.282	0.381	0.574	0.564
CD3^+^ cells (%)	71	75	64	68	75	64	66
CD4^+^ cells (%)	66	66	51	57	57	39	41
CD8^+^ cells (%)	4	6	11	10	16	21	21
CD56^+^CD16^+^ cells (%)	14	14	24	19	10	11	7
CD19^+^ cells (%)	15	9	10	12	14	24	26
CD4^+^FOXP3^+^ cells	Normal	Normal	−	−	−	−	−
T-cell proliferation after stimulation							
PHA	Normal	Normal	−	Normal	−	Normal	−
Anti-CD3 antibody	Normal	Normal	−	Normal	−	Normal	−
Immunoglobulins							
IgG (g/dL [6.0-16.0])	11.8	−	11.8	−	12.4	9.6	−
IgG_1_ (g/L [3.62-10.27])	7.48	−	−	−	−	−	−
IgG_2_ (g/L [0.81-4.72])	2.66	−	−	−	−	−	−
IgG_3_ (g/L [0.138-1.058])	0.420	−	−	−	−	−	−
IgG_4_ (g/L [0.049-1.085])	0.224	−	−	−	−	−	−
IgA (g/dL [0.80-4.00])	1.85	−	2.53	−	3.51	3.95	−
IgM (g/dL [0.25-2.00])	2.08	−	0.77	−	1.20	1.12	−
IgE (kU/L [0.5-120.0])	<2.0	−	195.0	−	24.4	157.0	−
Other antibodies							
ANA†	−ve	−ve	−ve	−ve	+ve†	+ve‡	−
dsDNA (IU/mL [0-50])	−	−ve	−	−	−ve	−ve	−
Rheumatoid factor (IU/mL [<20])	<15	<15	<15	−	122	−	−
Complement							
C3 (g/dL [0.75-1.65])	1.14	−	1.02	−	1.34	−	−
C4 (g/dL [0.12-0.40])	0.28	−	0.31	−	0.44	−	−

*−*, Not investigated; *ANA*, antinuclear antibody; *CMV*, cytomegalovirus; *dsDNA*, double-strand DNA; *ND*, not detected; *RNP*, ribonucleoprotein; *VCA*, viral capsid antigen; *+ve*, positive; *−ve*, negative; *VZV*, varicella zoster virus. Values in boldface are outside the reference ranges.

∗ On resampling 3 months later: CD4/CD8 ratio, 14.36; CD8, 0.092.

† Positive (homogenous: weak RNP antibody positive).

‡ Positive (nucleolar).

**Table E3:** Summary of key clinical findings associated with individual reported recessive *STIM1* mutations and summarized key clinical findings associated with dominant *STIM1* mutations

Feature	Recessive homozygous mutations						Dominant mutations	
Reference	Picard et al, 2009^E8^	Byun et al, 2010^E9^	Fuchs et al, 2012^E10^	Wang et al 2014^E11^	Schaballie et al, 2015^E12^	This study	Bohm et al, 2013^E13^	Morin et al, 2014^E14^
Individual (AR) or diagnosis (AD)	P^r^1, P^r^2, and P^r^3∗	P^r^4†	P^r^5 and P^r^6	P^r^7	P^r^8 and P^r^9	V2 and V3	Tubular aggregate myopathy‡	Stormorken syndrome‡
Predicted protein effect of mutation	No protein	No protein	p.429 R>C	p. 146A>V	p.165P>Q	p.74 L>P	All missense in the EF-hand	p.304 R>W
Age at last examination (y)	1, 5, 6, and 9	2	1.7 and 6	6	8 and 21	11 and 21	Various	Various
Immune deficiency	Life-threatening infections	Life-threatening infections	Life-threatening infections	History of frequent throat infections: no immunologic evaluation performed	Life-threatening infections	No persistent severe infection	NR	NR
Other immune dysregulation	AIHA ITP	AIHA	AIHA ITP	NR	Colitis, psoriasis	V3 transient ITP	NR	NR
Skeletal muscle	Developmental skeletal myopathy with hypotonia, profound	NR	Developmental skeletal myopathy with hypotonia, mild	NR	Developmental skeletal myopathy, profound	No abnormalities	Clinical myopathy except with 1 mutation Increased CK typical	Clinical myopathy Increased CK
Mydriasis	Yes	NR	Yes	NR	No	No	NC	Yes
Sweat glands	NC	NR	Anhidrosis	NR	Anhidrosis	Hypohidrosis	NC	NC
Dental enamel	Abnormal	NR	Abnormal	Abnormal	Abnormal	Abnormal	NC	NC
Died	P^r^1 died 9 y (during HSCT) P^r^2 died 1.5 y (encephalitis)	P^r^4 died 2 y (Kaposi sarcoma)	P^r^6 died 1.7 y (sepsis)	NR	NA	NA	NA	NA
Alive	P^r^3 alive at 6 y (HSCT at 1.3 y)	NA	P^r^5 alive (HSCT)	P^r^7 lost to follow-up at 5 y	P^r^8 and P^r^9 alive	V2 and V3 alive	All alive	All alive

*AIHA*, Autoimmune hemolytic anemia; *AD*, autosomal dominant; *AR*, autosomal recessive; *CK*, creatine kinase; *HSCT*, hematopoietic stem cell transplantation; *ITP*, idiopathic thrombocytopenic purpura; *NA*, not applicable; *NC*, no comment made; *NR*, comment made but feature not recognized.

∗ Mutation confirmed in P^r^1 and P^r^3; no DNA sample available for P^r^2.

† Mutation identified after death.

‡ A missense change reported in tubular aggregate myopathy and the missense change reported as the cause of Stormorken syndrome have also been identified as the causes of York platelet syndrome, which is characterized by myopathy and platelet abnormalities (Markello et al, 2015^E15^).

**Table I:** Summary of the main clinical and clinical immunologic features in subjects with either homozygous or heterozygous *STIM1* c.221T>C mutations

Feature	V:3	IV:4	IV:3	V:2
*STIM1* genotype	Homozygous c.221T>C	Heterozygous c.221T>C	Heterozygous c.221T>C	Homozygous c.221T>C
Age at evaluation (y)	18-21	45-48	41	9-12
Persistent infections	None	None	None	None
Other infections	Infancy: repeated chest infections but not thereafter	No reported issues	No reported issues	Infancy: chest, urinary tract, gastrointestinal tract, ear, and eye infections but not thereafter
Autoimmune disorder	Transient ITP aged 2.5 y	None	Sjogren syndrome	None
Lymphocytes				
Total (×10^9^/L [1.00-2.80])	1.50	2.30	2.64	2.34
CD4 (absolute; × 10^9^/L [0.300-1.400])	0.841	1.091	1.502	0.908
CD8 (absolute; × 10^9^/L [0.200-0.900])	**0.055**	0.236	0.415	0.488
CD4/CD8 (1.07-1.87)	**15.29**	**4.62**	**3.62**	1.86
NK (absolute; × 10^9^/L [0.090-0.600])	0.238	0.581	0.252	0.252
Immunization history	Full schedule without adverse events	Not assessed	Not assessed	Full schedule without adverse events
Musculoskeletal	Muscle bulk, tone, power, and reflexes normal; hypermobility in upper and lower limbs; CK normal	No issues evident; not formally examined	No issues evident; not formally examined	Muscle bulk, tone, power, and reflexes normal; hypermobility in upper and lower limbs; CK normal
Pupil reaction	Normal	Normal	Normal	Normal
Sweating	Diminished sweating recognized from infancy onward; insufficient sample for sweat test analysis	No reported issues	No reported issues	Diminished sweating recognized from infancy onward; reduced sweating on starch and iodine testing
Dental enamel	Generalized hypomineralized AI	Enamel within normal limits	Enamel within normal limits	Generalized hypomineralized AI
Development	Global development normal Height, 156 cm (<0.4th percentile) Weight, 40.3 kg (<0.4th percentile) Head circumference, 51.5 cm (<0.4th percentile)	Not assessed	Not assessed	Global development normal Height, 122 cm (0.4th-2nd percentile) Weight 24 kg (2nd-9th percentile) Head circumference, 50 cm (0.4th percentile)

Further details are presented in Table E1, Table E2, Table E3. Values in boldface are outside the reference ranges.

*CK*, Creatine kinase; *ITP*, idiopathic thrombocytopenic purpura.

## Methods

### Participating family

A consanguineous family of Pakistani heritage was reviewed in the clinical genetics clinic with regard to intolerance to warm environments and generalized dental enamel defects of both dentitions. Sample collection was performed after obtaining informed consent from the patients according to the principles of the Declaration of Helsinki and after local ethics approval. Detailed clinical evaluation was undertaken in appropriate clinical settings.

### Genetic mapping

DNA was extracted from blood by using standard procedures. DNA from the 2 affected subjects was genotyped with Affymetrix 6.0 SNP microarrays (Affymetrix, High Wycombe, United Kingdom), and regions of homozygosity were identified by using AutoSNPa software.^E1^ Linkage was confirmed by means of analysis with fluorescence-labeled polymorphic microsatellite markers on a genetic analyzer (3130*xl*Genetic Analyzer; Applied Biosystems, Warrington, United Kingdom) using genotyping software (GeneMapper version 4.0; Applied Biosystems). Linkage analyses were performed with LINKMAP and MLINK from the FASTLINK software package.^E2^

### Clonal and Sanger sequencing

We designed a SureSelect Target Enrichment Reagent (Agilent Technologies, Edinburgh, United Kingdom) targeting coding exons within the disease interval in parallel with the capture of disease intervals for 7 other unrelated disorders. The affected subject IV:N was sequenced with 80-nt reads on an Illumina (San Diego, Calif) GAIIx sequencer. Raw data were processed with the Illumina pipeline (version 1.3.4), and reads were aligned to the human reference sequence (hg19/GRCh37) by using Novoalign software (Novocraft Technologies, Selangor, Malaysia). Alignments were processed in the SAM/BAM format^E3^ with Picard and the Genome Analysis Toolkit (GATK)E4, E5 to correct alignments around indel sites and to mark potential PCR duplicates. Variants were called in the Variant Call Format by using the Unified Genotyper function of GATK. Filtering of common variation and prediction of functional consequences of variants were performed by using in-house scripts.

PCR products for *STIM1* exon 2 and *STK33* exon 3 were amplified and sequenced by using the primer pairs shown in Table E1. PCR product cleanup was performed with ExoSAP-IT (Affymetrix) before Sanger sequencing with the BigDye Terminator Cycle Sequencing Kit, version 3.1 (Applied Biosystems) and analysis on an ABI 3130XL DNA analyzer (Applied Biosystems).

### Flow cytometric analysis of calcium flux

PBMCs were labeled with Dulbecco modified Eagle medium containing 5 μmol/L Indo-1 for 45 minutes at 37°C and then washed and cooled on ice. Cells were incubated for 20 minutes on ice with 5 μg each of unconjugated CD16 (3G8) and CD3-PerCP (OKT3; BD Biosciences, San Jose, Calif) antibodies and costained for gating markers CD19 (SJ25C1) and CD56 (NCAM16.2; BD Biosciences). Cells were washed and resuspended in cold HBSS without calcium. Samples were warmed to 37°C and immediately collected on a UV laser equipped LSRII flow cytometer for 90 seconds and then spiked during collection with 1:100 goat anti-mouse antibody for a further 60 seconds (Jackson Laboratory, Bar Harbor, Me), followed by a 1:100 dilution of 200 mmol/L CaCl_2_ in PBS solution, and collected for a further 9 minutes. Alternatively, samples were stimulated with the calcium ionophore ionomycin at 500 ng/mL (Sigma-Aldrich, St Louis, Mo) or 1 μmol/L thapsigargin (Sigma-Aldrich) to deplete ER stores of calcium, thereby triggering SOCE and an intracellular calcium ([Ca^2+^]_i_) flux. Analysis was performed with FlowJo software (TreeStar, Ashland, Ore), calculating the ratio of calcium-bound to free Indo-1.

### NK cell responses

PBMCs were isolated from diluted blood by means of Ficoll separation, followed by NK cell purification by means of negative selection (with immunomagnetic reagents from Miltenyi Biotec, Bergicsch Gladbach, Germany). Isolated NK cells were stimulated with K562 tumor cells alone or in combination with 20 ng/mL IL-12/IL-18 (PeproTech, Rocky Hill, NJ; to maximize IFN-γ by tumor-stimulated cells) and incubated for 6 hours at 37°C with both GolgiStop and GolgiPlug (BD Biosciences). Cells were stained for the surface markers CD107a (clone; H4A3), CD56 (NCAM16.2), and CD3 (OKT3; BD Biosciences) before fixation for 15 minutes and permeabilization for 30 minutes with the AbD Serotec (Oxford, United Kingdom) intracellular staining kit. Cells were stained with anti–IFN-γ (B27) and collected on an LSR II flow cytometer and analyzed in DIVA software (BD Biosciences).

### STIM1 constructs for transfection studies

YFP-STIM1 (Addgene plasmid 18857) and the EF-hand mutant YFP-STIM1 (D76 A; Addgene plasmid 18859) constructs were provided by Tobias Meyer through Addgene (Cambridge, Mass). The ORAI1-CFP construct was provided by Anjana Rao (Addgene plasmid 19757). The L74P mutant YFP-STIM1 was produced by means of site-directed mutagenesis of the wild-type YFP-STIM1 plasmid by using the QuikChange II kit (Agilent Technologies, Santa Clara, Calif) per the manufacturer's instructions. The sequences of all 4 constructs were confirmed by means of Sanger sequencing, as above.

### TIRFM

HEK293 cells (LGC Standards, Middlesex, United Kingdom; ATCC no. CRL-1573) were grown on glass coverslips coated with poly-D-lysine and transfected with either wild-type YFP-STIM1, mutant YFP-STIM1 (D76 A), or mutant YFP-STIM1 (L74P) by using Lipofectamine 2000 (Invitrogen, Paisley, United Kingdom). Total internal reflection fluorescence (TIRF) imaging was performed on an inverted microscope (TE-2000E; Nikon) through a 60× oil-immersion lens (ApoTIRF 60×/1.49 numeric aperture; working distance, 0.12 mm; Nikon, Tokyo, Japan). Cells were maintained at 37°C and perfused with standard bath solution; ER store depletion was induced by 2 μmol/L thapsigargin. The plasma membrane was illuminated by using TIRF with a 488-nm argon laser (Prairie Technologies, Middleton, Wis), which was projected onto the specimen through the lens. Images were collected on an electron-multiplying CCD camera (DQC-FS, Nikon) by using NIS Elements imaging software, version 3.2 (Nikon), which was also used for analysis. Fluorescence intensities were background subtracted after acquisition and normalized to the initial intensity (F_0_).

### Calcium measurements in overexpressing cells

HEK293 cells were doubly transfected with ORAI1-CFP and either wild-type YFP-STIM1, mutant YFP-STIM1 (D76A), or mutant YFP-STIM1 (L74P). Twenty-four hours after transfection, cells expressing both CFP and YFP constructs were selected by using a Becton Dickinson FACSAria II cell sorter (BD Biosciences) and plated on glass coverslips. In each case basal [Ca^2+^]_i_ levels were recorded, after which Ca^2+^ was removed from the perfusate (replaced with 1 mmol/L ethyleneglycol-bis-(β-aminoethylether)-N,N,N′,N′-tetra-acetic acid), and new basal levels of [Ca^2+^]_i_ were determined. Cells were then exposed to CPA (100 μmol/L), and the resultant transient increases in [Ca^2+^]_i_ levels were measured for peak amplitude and integral. After washout of CPA, Ca^2+^ (2.5 mmol/L) was readmitted to the perfusate, and capacitative Ca^2+^ entry was quantified as the maximal increase in [Ca^2+^]_i_ observed. Data are presented as representative examples (see Fig E1, *B*) and mean ± SEM values (see Fig E1, *C*) determined from 12 control recordings, 12 recordings of D76A expressing mutants, and 13 recordings of L74P expressing mutants. Statistical significance was determined by means of ANOVA.

## Results

### Identification of a novel homozygous missense p.L74P change in STIM1

Autozygosity mapping identified a single region of homozygosity shared by both affected cousins on chromosome 11 between rs11606404 and rs3815045 (chr11:2,241,215-61,669,946, hg19). Multipoint linkage analysis of markers D11S921, D11S899, D11S915, and D11S4949 against disease by using LINKMAP results in a maximum LOD score of 3.06 at marker D11S899. On merging of overlapping exon intervals, the disease interval contained 3,838 RefSeq coding regions comprising 751,450 bp, 3,784 (739,189 bp or 98.4%) of which could be targeted while avoiding designing baits over repeat masked regions. After target enrichment, sequencing, alignment, and postprocessing, 94.6% of targeted bases were covered by 5 or more nonduplicate reads with a minimum Phred-like base quality score of 17 and minimum read mapping quality of 20.

A total of 526 variants passing standard GATK filters were identified within 20 bp of a coding exon within the disease locus. Variants were removed if present in dbSNP129 or in later versions of dbSNP with a minor allele frequency of 1% or greater, if present in other samples sequenced locally (n = 31), or, in the case of missense variants, if predicted to be benign by using PolyPhen-2.^E6^ After these filtering steps, only 3 homozygous variants remained that might be predicted to alter gene function. The first of these (NM_152316: c.3G>C) was considered unlikely to be pathogenic despite altering the initiation codon of *ARL14EP* because of the presence of another in-frame initiation codon immediately adjacent to the mutated codon and lack of conservation of the first of these ATG codons in mammals. Of the remaining 2 changes, a missense mutation in *STK33* (NM_030906: c.146G>A; p.G49D) was found in 4 of 96 ethnically matched control samples, whereas a missense mutation in *STIM1* (NM_003156: c.221T>C; p.L74P) was excluded in 192 ethnically matched control samples and found to segregate as expected for a recessively inherited disease within the family. Subsequent interrogation of the Exome Aggregation Consortium database showed that although the *STK33* variant was present at a frequency of 1.49% in subjects of South Asian ancestry, the *STIM1* variant was not detected at all in the cohort of 60,706 subjects (Exome Aggregation Consortium, Cambridge, Mass; http://exac.broadinstitute.org; accessed February 2015).

Accordingly, the homozygous c.221T>C; p.L74P mutation identified in *STIM1* was therefore considered to be the cause of the observed phenotype based on genetic data and the phenotypic overlap with previously reported recessive *STIM1* and *ORAI1* mutations.
